# The integration of Gβ and MAPK signaling cascade in zygote development

**DOI:** 10.1038/s41598-017-08230-4

**Published:** 2017-08-18

**Authors:** Guo-Liang Yuan, Hong-Ju Li, Wei-Cai Yang

**Affiliations:** 1 State Key Laboratory of Molecular and Developmental Biology, Institute of Genetics and Developmental Biology, Chinese Academy of Sciences, Beijing, 100101 China; 2 Beijing Engineering and Technique Research Center for Hybrid Wheat, Beijing Academy of Agriculture and Forestry, Beijing, 100097 China

## Abstract

Cells respond to many signals with a limited number of signaling components. Heterotrimeric G proteins and MAPK cascades are universally used by eukaryotic cells to transduce signals in various developmental processes or stress responses by activating different effectors. MAPK cascade is integrated with G proteins by scaffold protein during plant immunity. However, the molecular relationship between G proteins and MAPK modules in plant development is still unclear. In this study, we demonstrate that Arabidopsis Gβ protein AGB1 interacts with MPK3 and 6, MKK4 and 5, as well as the regulatory domains of YODA (YDA), the upstream MEKK of MKK4/5. Remarkably, YDA interacts with the plasma membrane associated SHORT SUSPENSOR (SSP) through its N- and C-terminal region *in vitro* and *in vivo*. Additionally, genetic analysis shows that AGB1 functions together with MPK3/6 signaling cascade during the asymmetric division of the zygote. These data indicate that Gβ may function likely as a scaffold, through direct physical interaction with the components of the MPK signaling module in plant development. Our results provide new insights into the molecular functions of G protein and will advance the understanding of the complex mechanism of kinase signaling cascades.

## Introduction

Heterotrimeric G proteins transmit numerous and diverse extracellular cues by coupling with the plasma membrane-localized receptors and different effectors in eukaryotic cells. In contrast to metazoans, the repertoire of genes encoding G proteins subunits in plants is small. The *Arabidopsis thaliana* genome encodes one prototypical Gα (GPA1), one Gβ (AGB1), three Gγ subunits (AGG1, AGG2 and AGG3) and one regulator of G-protein signaling (AtRGS1) protein^1^. In Arabidopsis and rice, heterotrimeric G proteins play diverse roles in a variety of processes, such as hormone regulation, drought stress, pathogenesis, and development^2^, and they often serve as a converging point of different signaling pathways triggered by distinct receptors^3^. Genetic and biochemical evidences implicated that G proteins likely integrate signaling pathways as a variable resistor to control the output of diverse signal information^4^. However, the molecular basis of G proteins in plant signaling is yet to be determined.

Mitogen-activated protein kinase (MAPK) cascade is evolutionarily conserved and mediates diverse cellular responses to a variety of extra- and intracellular stimuli in eukaryotes^5^. Phosphorylation activation of MAPKs is executed by MAPK kinases (MAPKKs or MEKs), which are phosphorylated by MAPKK kinases (MAPKKKs or MEKKs). One main question in MPK cascade signaling is how the large signaling diversity is achieved by similar or the same modules. One pivotal way to regulate signaling modules is through scaffold proteins^6, 7^. In animals and fungi, MPK modules bind different scaffold proteins which promote signaling efficiency and/or specificity^6, 8–10^. In mammals, the WD40 repeat protein Receptor for Activated C Kinase 1 (RACK1) has been established as a scaffold protein by interacting with a range of proteins in global control of gene transcription, translation, and ribosome assembly and activation^11–13^. Recently, it was shown that RACK1 interacts with AGB1 and functions as a dynamic scaffold of MEKK1-MKK5-MPK3/6 cascade during plant immune response^14^. It has been proposed that the diverse roles of Gβ may be conferred by its binding to distinct WD40 repeat-containing proteins which mediate diverse cellular processes in animals^15^. Similar to RACK1, Gβ itself contain seven WD40 repeats which adopt a circular β-bladed propeller structure^16^. This unique ternary structure confers its property to interact with different proteins, while the molecular mechanics of Gβ in diverse signaling is still unclear in plants.

In plants, the zygote elongates largely after fertilization and before the first asymmetric cell division which is essential for the following embryo development. This process is regulated by a kinase-dependent pathway. Loss of *YDA*, encoding a MAPKKK, suppresses zygote elongation and causes shortened suspensor cells and embryo lethality^17^. Loss of *SSP*, which encodes a sperm-derived kinase, causes a similar phenotype to *yoda* ^18^. This suggests that SSP likely activates YDA and initiates zygote elongation although the mechanism is unknown. Knock-down of *MKK4* and *MKK5*, which activates MPK3/6, causes developmentally-arrested embryo^19^. The *mpk3 mpk6* double mutant suppresses suspensor formation and ovule development^19^. YDA has been verified to be an upstream MAPKKK of MKK4/MKK5-MPK3/MPK6 module in stomata development and patterning^20^. Recently, we found that a leucine-rich repeat receptor-like kinase ZAR1 regulates zygote elongation and asymmetric division through AGB1 and SSP^21^. In these contexts, the kinases-governed zygote development appears dependent on a ZAR1-SSP-YDA-MKK4/5-MPK3/6 signaling cascade. In this pathway, how the signals are relayed and how G proteins are integrated are still unclear.

In this study, we provide biochemical and genetic evidences that AGB1 directly interacts with MPK3/6. AGB1, but not GPA1, interacts with MKK4/5. AGB1 and SSP interact with the extended N- and C-terminal domains but not the kinase domain of YDA. Further genetics study also confirmed the genetic interaction between *MPK3/6* and *AGB1* during zygote and fruit development. These data support the model that AGB1 acts as a scaffold for the MAPK signaling cascade during Arabidopsis development.

## Results

### Heterotrimeric G protein subunits physically interact with MPK3 and MPK6

**Figure 1:**
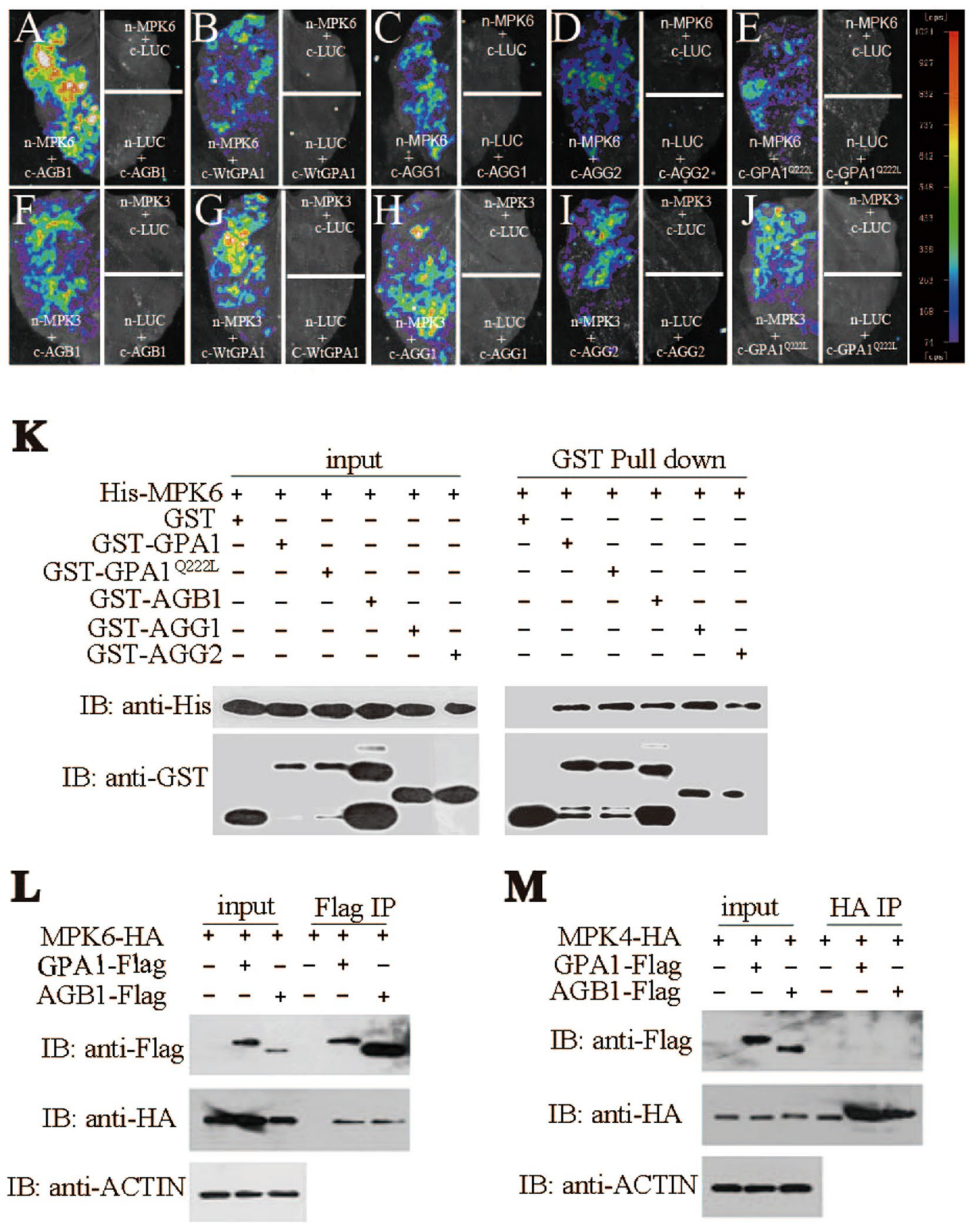
AGB1, GPA1, AGG1 and AGG2 interact with MPK6 and MPK3. (**A**–**J**) Tobacco leaves co-infiltrated with agrobacterium containing *35S*-driven split luciferase (LUC) constructs as indicated were photographed with a charge-coupled device camera. Each image is representative of three images in three independent experiments. (**A**–**E**) MPK6-nLUC interacts with cLUC-AGB1 (**A**), cLUC-WtGPA1 (**B**), cLUC-AGG1(**C**), cLUC-AGG2 (**D**), cLUC-GPA1^Q222L^ (**E**), respectively. (**F**–**J**) MPK3-nLUC interacts with cLUC-AGB1 (**F**), cLUC-WtGPA1 (**G**), cLUC-AGG1 (**H**), cLUC-AGG2 (**I**), cLUC-GPA1^Q222L^ (**J**), respectively. The pseudocolor bar shows the relative range of luminescence intensity in images. Pull-down assay shows that AGB1, WtGPA1, GPA1^Q222L^, AGG1 and AGG2 interact with MPK6 (**K**), respectively. AGB1 and GPA1 interact with MPK6 (**L**), but not MPK4 (**M**) by Co-IP assay. Full blots are shown in Supplemental Data.

**Figure 2:**
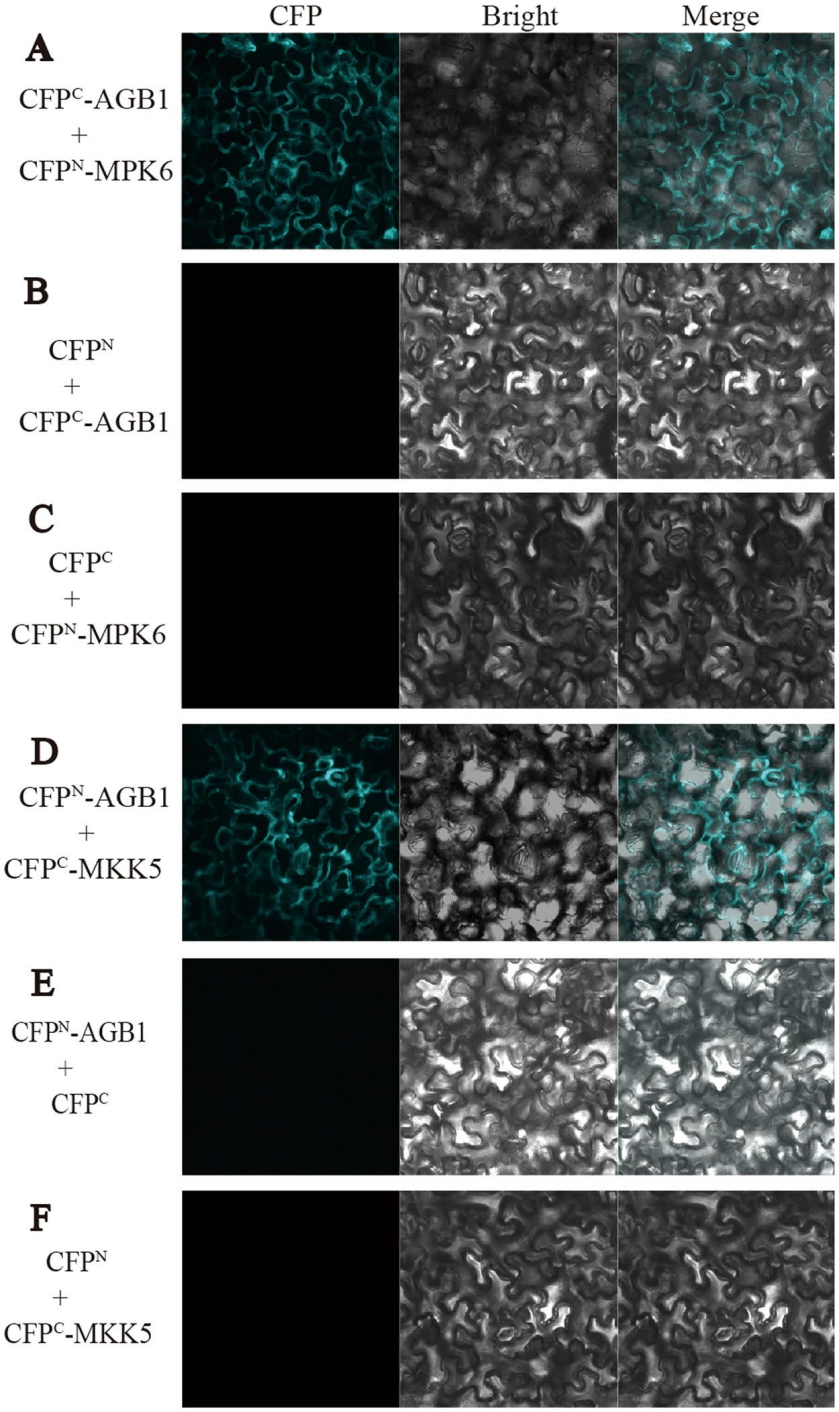
Bimolecular fluorescence complementation analysis showing the interaction between AGB1, MPK6 and MKK5. Images of tobacco leaf sections expressing CFP^C^-AGB1 and CFP^N^-MPK6 (**A**), CFP^N^-AGB1 and CFP^C^-MKK5 (**D**) exhibit fluorescence, but not CFP^C^-AGB1 and CFP^N^ (**B**), CFP^C^ and CFP^N^-MPK6(**C**), CFP^N^-AGB1 and CFP^C^ (**E**), or CFP^N^ and CFP^C^-MKK5 (**F**). Each image is a representative of three images in three independent experiments.

Since both AGB1 and YDA-MKK4/MKK5-MPK3/MPK6 module play roles in plant architecture, zygote and embryo development^19, 21–23^, we are curious about whether heterotrimeric G proteins and MPK3/6 signaling module interact biochemically and genetically. To answer this question, we first examined the interaction of GPA1, AGB1, AGG1 and AGG2 with MPK3 and MPK6 *in vivo* by a firefly luciferase complementation imaging assay in tobacco leaves^24^. As shown in Fig. 1A–J, combinations of MPK6-nLUC or MPK3-nLUC with cLUC-AGB1, cLUC-AGG1, cLUC-AGG2, cLUC-GPA1 and cLUC-GPA1^Q222L^ (the constitutively active form of GPA1) generate strong luciferase activity signals, indicating that GPA1, AGB1, AGG1 and AGG2 could interact with MPK6 and MPK3. Pull-down assay with the purified epitope-tagged proteins confirmed the interaction between MPK6 and different G protein subunits (Fig. 1K). These results suggest that the interaction of GPA1 with MPK3/6 is independent of its GTPase activity. Coimmunoprecipitation (Co-IP) result further shows that GPA1 and AGB1 interact selectively with MPK6, but not with MPK4 *in vivo* (Fig. 1L,M), indicating that the interaction is specific. In addition, through bimolecular fluorescence complementation (BiFC) assay in tobacco leaf, we further confirmed the interaction between MPK6 with AGB1 (Fig. 2A–C). Together, these results suggest that each component of the heterotrimeric G proteins physically interact with MPK3/6 both *in vitro* and *in vivo*.

### AGB1 but not GPA1 interacts with MKK4 and MKK5

**Figure 3:**
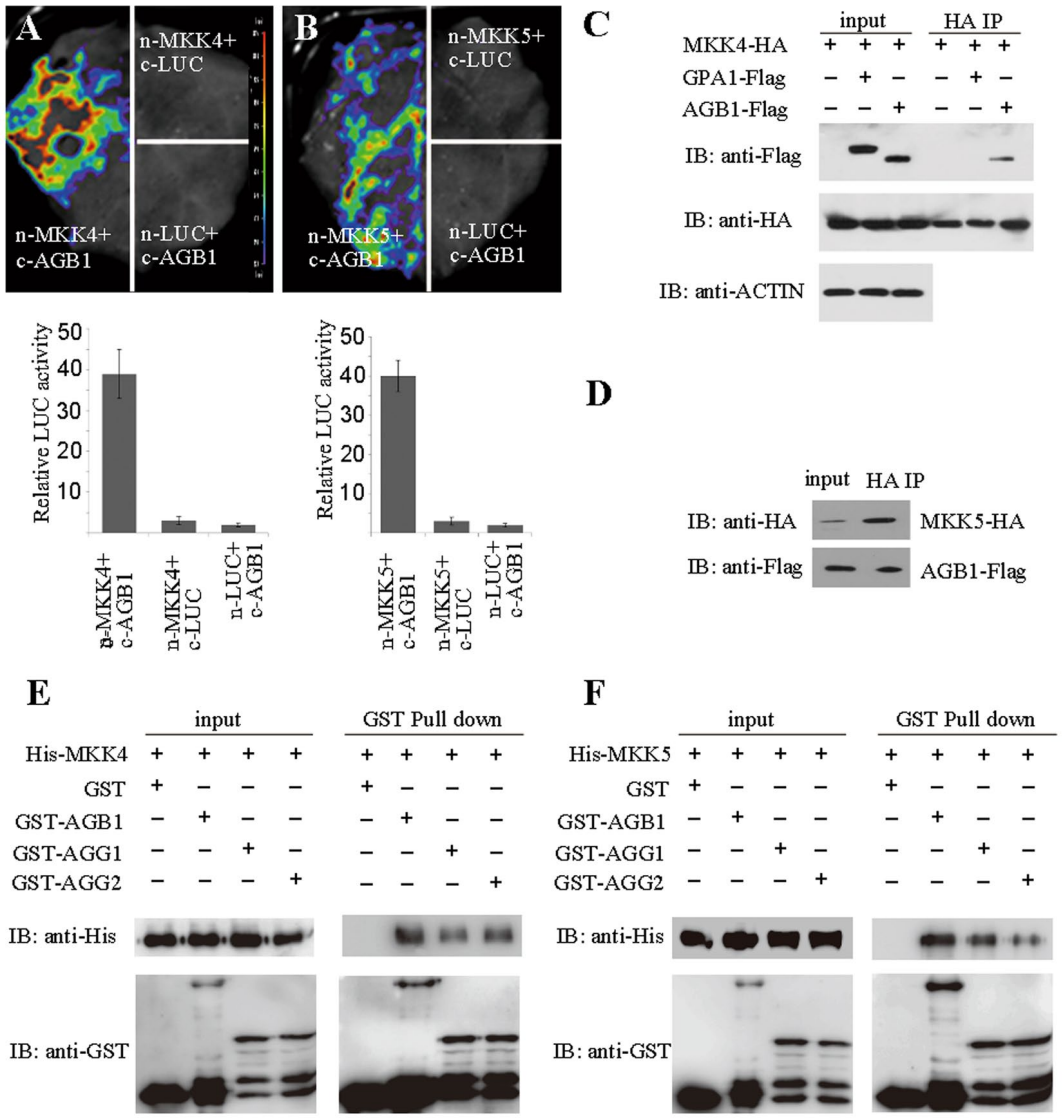
AGB1 interact with MKK4 and MKK5. (**A**,**B**) Tobacco leaves co-infiltrated with agrobacterium containing *35S*-driven construct pairs as indicated were photographed with a charge-coupled device camera. AGB1-cLUC interacts with nLUC-MKK4 (**A**) and nLUC-MKK5 (**B**). The upper lane in (**A**) and (**B**), luminescence images; the lower lane in (**A**) and (**B**), intensity quantification of the upper lane. The pseudocolor bar shows the relative range of luminescence intensity in images. (**C**) Co-IP assay in Arabidopsis protoplasts shows that AGB1 interacts with MKK4 (**C**) and MKK5 (**D**), but not GPA1. Pull-down assay shows that AGB1, AGG1 and AGG2 interact with MKK4 (**E**) and MKK5 (**F**). Full blots are shown in Supplemental Data.

MKK4/5 relay the signal from YDA to MPK3/6 in plant architecture regulation. *MKK4/MKK5* knock-down plants phenocopy *agb1* in controlling fruit length^23^. To elucidate whether AGB1 interacts with MKK4/5, we performed biochemical analysis. Direct interaction between MKK4/5 and AGB1 was detected in tobacco leaves using BiFC (Fig. 2D–F) and firefly luciferase complementation imaging (Fig. 3A and B) assay, respectively. Co-IP assay with Arabidopsis protoplasts shows that AGB1 interacts with MKK4 and MKK5, while GPA1 does not (Fig. 3C and D). Furthermore, pull-down assay also demonstrated that AGB1 interacts with MKK4 and MKK5 (Fig. 3E and F), while GPA1 does not (Fig. S1). These results suggest a directly physical interaction between AGB1 and MKK4/5 both *in vitro* and *in vivo*.

### AGB1 and SSP interact with the regulatory domains of YDA

**Figure 4:**
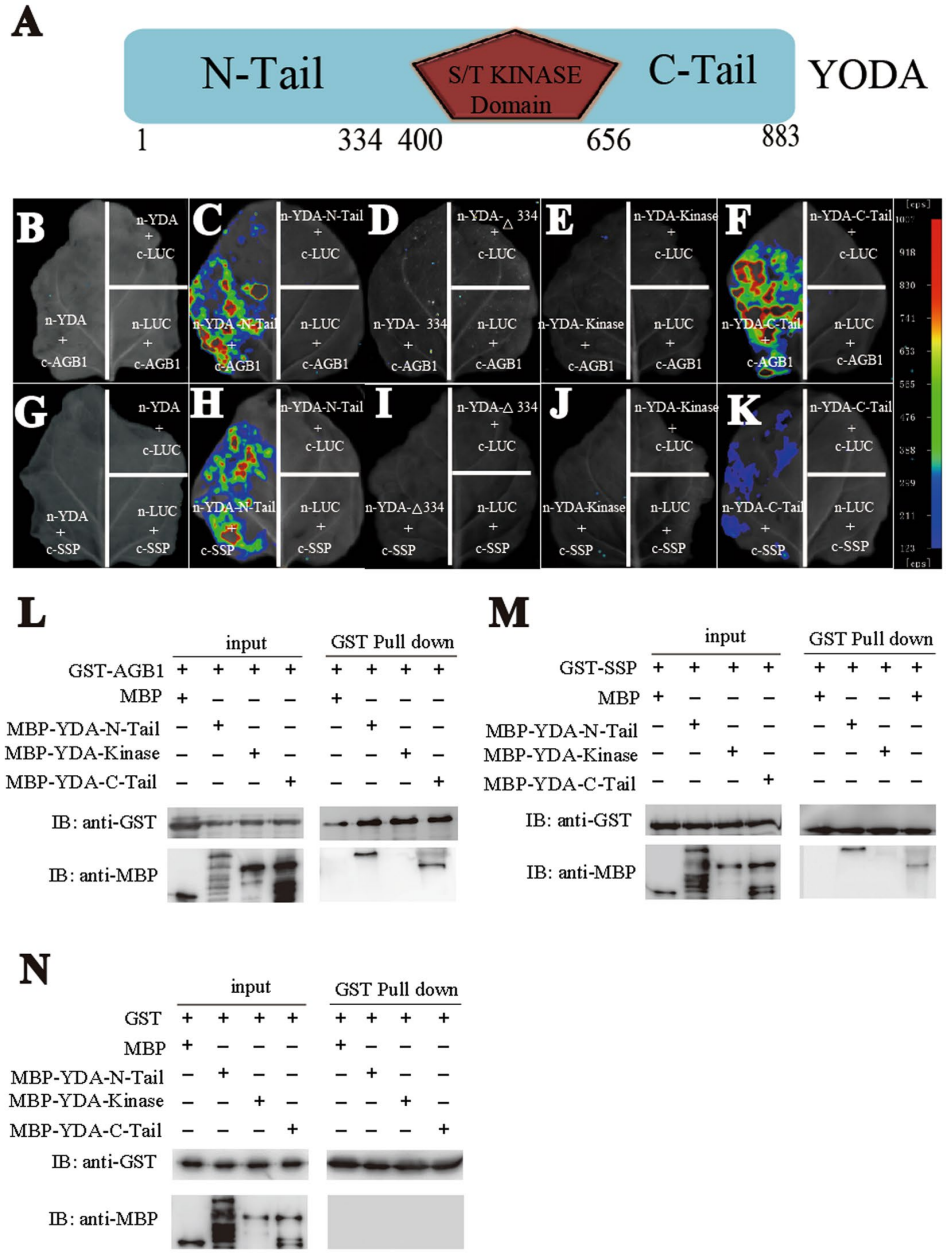
AGB1 and SSP physically interact with the N- and C-termial of YODA by luciferase complementation assay and pull-down assay. (**A**) Protein structure of YODA. N-Tail, kinase, C-Tail and Δ334 (334–883aa) are indicated in the diagram. (**B**–**F**) Tobacco leaves co-infiltrated with agrobacterium containing *35S*-driven construct pairs as indicated were photographed with a charge-coupled device camera. (**B**–**F**) cLUC-AGB1 interacts with nLUC-YDA-N-Tail (**C**) and nLUC-YDA-C-Tail (**F**), but not nLUC-YDA (**B**), nLUC- YDA-Kinase (**E**) or nLUC-YDA-Δ334 (**D**), respectively. (**G**–**K**) cLUC-SSP interacts with nLUC-YDA-N-Tail (**H**) and nLUC-YDA-C-Tail (**K**), but not nLUC-YDA (**G**), nLUC-YDA-Kinase (**J**) or nLUC-YDA-Δ334 (**I**), respectively. The pseudocolor bar shows the relative range of luminescence intensity in images. Pull-down assay shows that AGB1 (**L**) and SSP (**M**) interact with the N-Tail and C-Tail of YODA, but not the kinase domain. GST does not interact with the N-Tail, kinase domain or C-Tail of YODA (**N**). Full blots are shown in Supplemental Data.

Then we speculated that if AGB1 functions as a scaffold protein for the MPK cascade, AGB1 might also interact with the MAPKKK. Based on this hypothesis, we examined the interaction between AGB1 and YDA. YDA contains an N-terminal, a kinase-active and a C-terminal domain (Fig. 4A). The N-terminal domain is a negative regulatory domain which inhibits the kinase activity of YDA, while the C-terminal is required for the full activity of the kinase domain^17^. Firefly luciferase complementation assay shows that AGB1 interacts with both the N- and C-terminal of YDA, but not with the full-length protein (Fig. 4B–F). Consistently, pull-down assay also shows that AGB1 interacts with the N- and C-terminal of YDA, but not the kinase domain (Fig. 4L). Both constitutive activation and loss-of-function of *YDA* are detrimental to plants^17^, suggesting the physiological importance of the spatiotemporal modulation of its kinase activity. Furthermore, SSP genetically activates YDA after the zygote formation but the molecular mechanism is unknown. We showed that SSP strongly interacts with the N-terminal and weakly with the C-terminal of YDA, but not with the full-length protein (Fig. 4G–K). In addition, pull-down assay further shows that SSP interacts with the N- and C-terminal of YDA, respectively, but not the kinase domain (Fig. 4M and N). This specific interaction of AGB1 and SSP with the regulatory domains of YDA indicates that AGB1 and SSP may be directly involved in the signaling of YDA. And these results confirm the speculation that AGB1 interacts with different layers of MPK signaling cascade.

### *AGB1* and *MPK6* function together in zygote development

**Figure 5:**
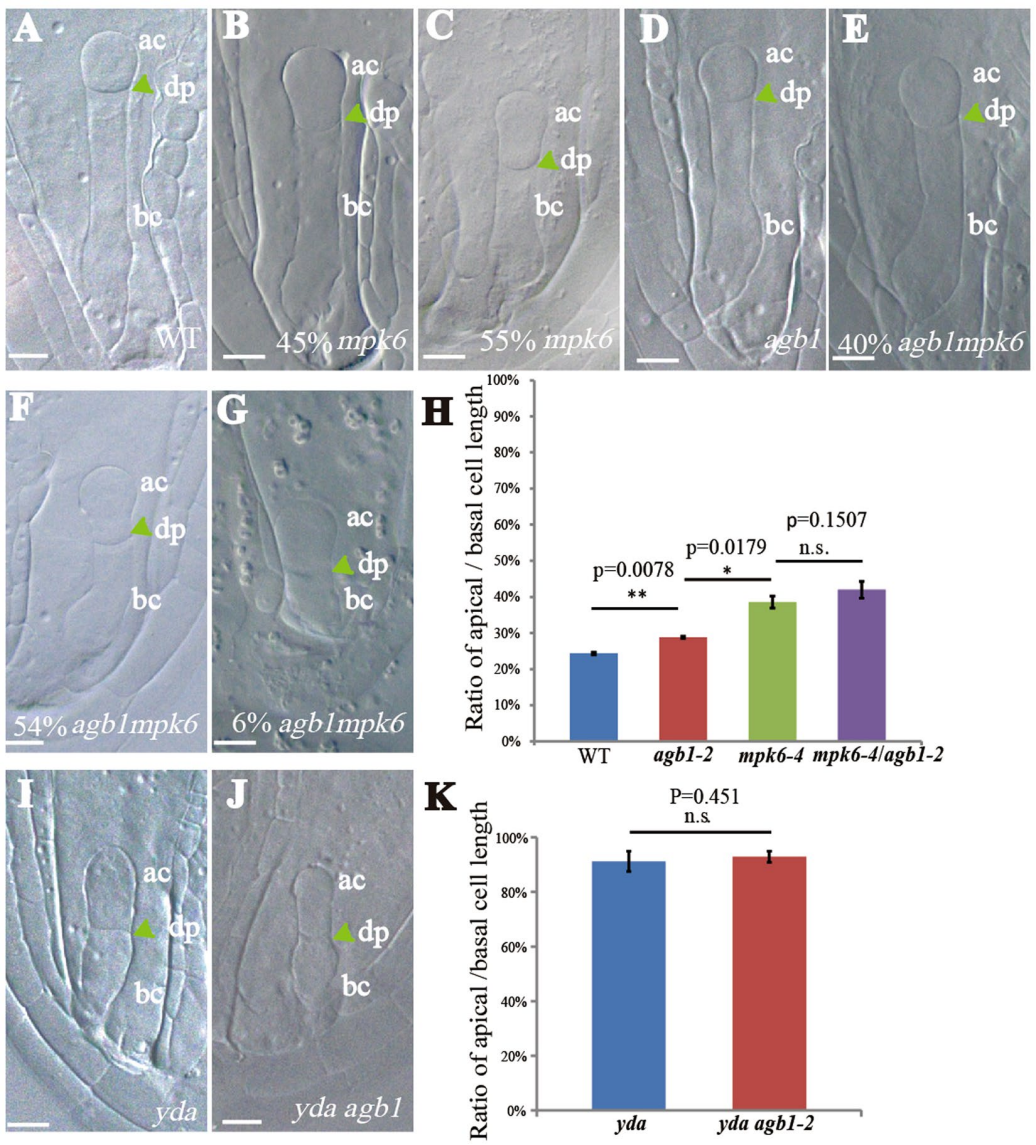
Zygotic phenotype of *mpk6, yda* and *agb1* mutants. (**A**–**G**) Representative images of the two-cell stage zygote in the wild type (**A**) and *mpk6-4* (**B** and **C**), *agb1*-*2* (**D**), *yda-8* (**I**) and *m6*-*4 agb1*-*2* (**E** to **G**), *yda*-*8 agb1*-*2* (**J**) mutants. (**H** and **K**) Statistical ratio of apical/basal cell length. Averages ( ± SE, n ≥ 30 per genotype) were determined in three independent experiments. ac, apical cell; bc, basal cell; dp, division plane. Students’ *t* test. p* < 0.05, p* < 0.01. n.s. no significance. Bars = 10 μm.

To investigate the genetic relation between *mpk6* and *agb1*, the early embryonic phenotype was studied. In *mpk6* homozygotes, two categories of phenotypes based on the length of the daughter cells of the zygote were observed: Category I in which the total length of apical and basal cell is similar or slightly shorter than the wild-type (45%, Fig. 5A and B) and Category II in which the length is significantly shortened (55%, Fig. 5C). Similarly, as previously reported^21^, *agb1* causes shortened basal cell leading to an increased ratio of the apical to basal cell length (Fig. 5D). In the *agb1 mpk6* double mutant, 60% embryos show the shortened phenotype (Category II) (Fig. 5E–G). Furthermore, we measured the ratio of the apical/basal cell length of all the sibling embryos in *mpk6-4*, *agb1-2* and *agb1-*2 *mpk6-4*. The results show that the average ratio for *agb1* and *mpk6* is higher than the wild-type, while the average ratio for *agb1 mpk6* is of no significance to that of the *mpk6* (Fig. 5H). However, 6% of *agb1 mpk6* zygotes exhibit severe reduced elongation which is not seen in single mutant, and display almost symmetric cell division compared with that in *mpk6* (Fig. 5G). It is not known how such a phenotypic variation takes place. MPK6 appears more important than AGB1 in controlling the zygotic elongation and division since *mpk6* exhibits a stronger phenotype than *agb1*, although they physically interact. It is possible that the scaffold role of AGB1 is dispensable for the MPK cascade which can assemble with less efficiency without AGB1. Alternatively, other scaffold proteins take over AGB1’s role in the absence of AGB1, or other MPKs such as MPK3 takes over when MPK6 is absent. Indeed, *MPK6* and *MPK3* were reported to function redundantly in suspensor formation^19^. Furthermore, the suspensor cells of *yda* are also shortened due to defective zygote elongation and symmetric division^21^. It is noticeable that the increased ratio of the apical to basal cell length of *yda* is more pronounced. To determine the genetic relationship between *AGB1* and *YDA*, *agb1-*2 *yda* double mutant was also constructed. The result shows that the ratio for *agb1-*2 *yda* is the same as *yda* (Fig. 5I–K), indicating that *yda* is epistatic to *agb1*. Together, we conclude that AGB1 genetically acts in the same pathway with the YDA-MPK6/3 signaling cascade during zygote elongation and division.

### Genetic relationship between G protein subunits and MPK3/6 in fruit development

**Figure 6:**
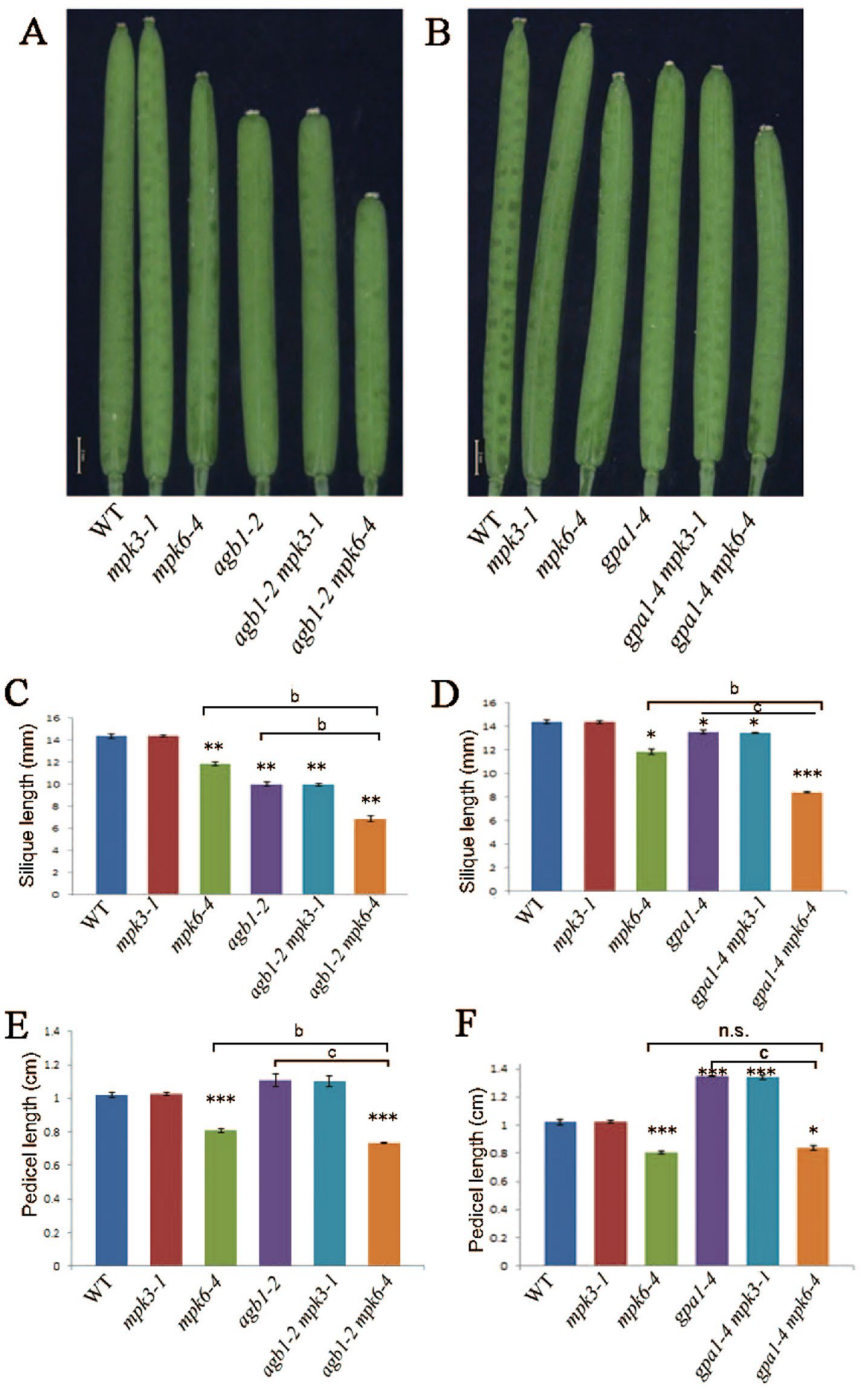
Length of siliques and pedicels of *agb1*, *gpa1*, *mpk3* and *mpk6*. (**A**) Siliques of wild-type plants (Col-0), *mpk3-1*, *mpk6-4, agb1-2* and double mutant *agb1-2 mpk3-1*, *agb1-2 mpk6-4*. (**B**) Siliques of wild-type plants (Col-0), *mpk3-1*, *mpk6-4, gpa1-4* and double mutant *gpa1-4 mpk3-1*, *gpa1-4 mpk6-4*. Bar, 1 mm. (**C** and **D**) Statistics of silique length of wild-type plants (Col-0), *mpk3-1*, *mpk6-4*, *agb1-2*, *gpa1-4* and double mutant *agb1-2 mpk3-1*, *agb1-2 mpk6-4*, *gpa1-4 mpk3-1*, *gpa1-4 mpk6-4*. (**E** and **F**) Statistics of pedicel length of genotype mentioned above. Averages (±SE, n ≥ 30 per genotype) were determined in three independent experiments. Different letters indicate significantly different values (b, p < 0.01; c, p < 0.001; ANOVA, Tukey test). Asterisks indicate a significant difference between a given genotype and the wild type (*p < 0.05, **p < 0.01, ***p < 0.001, Student’s *t* test). n.s. no significance.

G proteins and the MPK3/6 cascade were reported to regulate fruit length^22, 23^. Thus, we further examined the genetic interaction between G proteins and MPK3/6 during fruit development. Previous studies showed that null mutant of *AGB1* exhibits pleiotropic developmental defects, including shorter siliques^25^. To confirm the physiological relevance of the direct interaction of AGB1 and GPA1 with the MPK cascade in silique development, we analyzed the silique length of *agb1-*2 *mpk6-4*, *agb1-*2 *mpk3-1*, *gpa1-4 mpk6-4* and *gpa1-4 mpk3-1*. The *agb1-2* mutant exhibited shorter siliques than the wild-type as previously reported^22^, but double mutant *agb1-*2 *mpk6-4* displayed much shorter siliques than *agb1-2*, only 60% of the wild-type (Fig. 6A and C). The *agb1-2 mpk3-1* exhibits the same silique length as *agb1-2. mpk3-1* does not exhibit shorter siliques compared to the wild-type, while *gpa1-4* and *mpk6* displayed clearly obvious shortened siliques (Fig. 6A–D). This data indicate that MPK6 plays a role in fruit length which is enhanced by AGB1. Intriguingly, the shortened silique phenotype of *mpk6* is also exaggerated by *gpa1*, as *gpa1-4 mpk3-1* exhibits the same silique length as *gpa1*, while *gpa1-4 mpk6-4* displays much shorter siliques than *mpk6* (Fig. 6B,D). This result suggests that GPA1 also plays a role in MPK signaling cascade during fruit development.

Except for fruit length, pedicel length is also regulated by the MPK3/6 cascade^23^. Deletion of *AGB1* mildly exacerbates the shortened pedicel of *mpk6*, although *agb1* mutant does not show shortened pedicels (Fig. 6E). Pedicels of *gpa1* are longer than the wild-type, while *gpa1 mpk6* pedicels are comparable to *mpk6* (Fig. 6F), implying that in contrast to AGB1, GPA1 likely function in a different pathway in pedicel development.

Together, we conclude that MPK6 and AGB1 play positive roles in controlling zygote and fruit development. The additive effect of *mpk6* and *agb1* is possibly due to the functional redundancy of MPK3 and MPK6, as well as AGB1 and other WD40-repeat proteins which has been reported previously^19, 23, 26^. GPA1 also positively promotes silique development, but plays a negative role in pedicel length. GPA1 does not associate with MPKK4/5 as we suggested and dissociates with Gβγ in plants^27^, implying that GPA1 may not function as a scaffold of MPK cascade as AGB1.

## Discussions

In this study, we demonstrated the biochemical and genetic interactions between heterotrimeric G proteins and the MPK signaling components. Our results suggest that AGB1 and the MPK cascade may function together during zygote and fruit development.

Proper development of the zygote is dependent on receptor-like kinases, plasma membrane associated kinases and the MAPK signaling pathway, very similar to the early fertilization process^28^. Direct hierarchical interaction between components of the ZAR1-SSP-YDA-MKK4/5-MPK3/6 signaling cascade has been genetically and biochemically revealed, but SSP-YDA interaction was only implicated by genetic analysis. AGB1 plays a positive role in regulating the kinase activity of ZAR1 through direct interaction^21^. In this study, we showed that AGB1 genetically and biochemically interact with YDA, MKK4/5, and MPK3/6. And strikingly, we detected the direct interaction between SSP and YDA. These findings potentiate AGB1 as a fine-tuning scaffold protein in the kinase signaling module.

We show that AGB1 could interact with MPK3/MPK6, MKK4/MKK5 and regulatory domains of YDA. The biological significance of this scaffolding is still unclear. One possible effect is to recruiting different components to promote signal transduction. However, we found that AGB1 does not promote the interaction between MKK4/5 and MPK3/6 when AGB1 was co-expressed with MKK5 and MPK6 in Arabidopsis protoplasts (Fig. S2). One likely reason is that the promoting effect needs upstream extracellular signals from the neighboring cells or the environment. Another possible effect is to localize the signaling cascade at specific cellular compartment. ZAR1 and SSP are localized on the plasma membrane, while the MPK kinases are distributed extensively throughout the cell. AGB1 appears to be membrane-associated by lipid modification which supports it as a signaling component to transduce signal from outside to inside the cell. Third, AGB1 may affect the kinase activity of MPK3/6 to their substrates in the cellular responses. AGB1 has been shown to promote the kinase activity of ZAR1^23^. Forth, the scaffolding of a signal module could insulate related pathways from cross-talk or improve signaling efficiency or fidelity.

In metazoans and yeasts, scaffold proteins not only bring MAPK components together to enhance specificity and accelerate their activation, but also sequester MAPK modules to distinct subcellular locations in response to different types of stimuli^29^. Interestingly, AGB1 interacts with RACK1 that recruits MPK signaling cascades in plant immunity^14^. AGB1 has an N-terminal helix motif and a C-terminal WD40-repeat domain that forms a seven-bladed propeller structure^30^. Combining with our results, AGB1 and other WD40-repeat containing proteins, can function as scaffold proteins by forming dimers or even oligomers to integrate signals from different cellular processes in plants. Such dimerization mechanism of scaffold proteins is also utilized by RACK1 in mammals via interacting with Gβ^15^. Although RACK1 is not a Gβ as it does not bind GPA1 and lacks the N-terminal Gγ-binding motif, RACK1 shares similar WD40-repeat domains^31^. Interestingly, *rack1 agb1* double mutation causes more severe growth defects than both single mutant, indicating that RACK1 performs overlapping roles with AGB1 during plant development^26^. Furthermore, single MPK cascade can utilize multiple scaffold proteins which can render a wide-range of signaling potentials and fine-tuning^7^. In plants, AGB1 and RACK1 may function as a scaffold complex in the MPK signaling cascade, although the genetic relationship between RACK1 and MPK cascade components during development is yet to be determined. Our results support the hypothesis that Gβ, similar to RACK1, also functions as a scaffold protein for multiple protein kinases to enhances the robustness of plants development.

## Materials and Methods

### Plant materials and growth conditions

*Arabidopsis thaliana* ecotype Columbia-0 (Col-0), the T-DNA insertion lines CS6536 (*agb1-2*), SALK_016750 (*mpk6-4*), SALK_100651 (*mpk3-1*) and SALK_001846 (*gpa1-4*), Salk_105078 C (*yda-10*) were obtained from ABRC stock center (http://www.arabidopsis.org). Plants were grown in an air-conditioned room at 22 °C under a 16-hlight/8-h-dark cycle.

### Firefly luciferase complementation imaging assay

To generate MPK6-nLUC, MPK3-nLUC, MKK5- nLUC and MKK4- nLUC, YDA-nLUC, YDA-N-Tail-nLUC, YDA-C-Tail-NLuc, and YDA-Kinase-nLUC, the corresponding coding sequences were subcloned into pCAMBIA-nLUC^32^. To generate cLUC-AGB1, cLUC-GPA1, cLUC-AGG1, cLUC-AGG2, and cLUC-SSP, the corresponding coding sequences were subcloned into pCAMBIA-cLUC. The constructs were transformed into agrobacterium strain GV3101. Bacterial suspensions in MgCl_2_ were infiltrated into leaves of 7-week-old *Nicotiana benthamiana* plants using a needleless syringe. After infiltration, plants were grown in 16 h light/8 h darkness for 3 days at 22 °C. Images were captured by a low-light cooled charge-coupled device imaging apparatus (NightOWL II LB983).

### BiFC assay

To generate CFP^N^-AGB1 and CFP^N^-MPK6, the corresponding coding sequences were subcloned into pSPYNE^33^. To produce CFP^C^-AGB1 and CFP^C^-MKK5, the corresponding coding sequences were subcloned into pSPYCE. The constructs were then transformed into agrobacterium strain GV3101. Bacterial suspensions were infiltrated into leaves of 7-week-old *N. benthamiana* plants using a needleless syringe. After infiltration, plants were grown in 16 h light/8 h darkness for 3 days at 22 °C. For microscopic analyses, leaf discs were cut for imaging of BiFC signal by confocal laser scanning microscopy (LSM510META, Zeiss).

### Pull-down assay

Fusion proteins were expressed in *E. coli* and purified using glutathione agarose beads (GE Healthcare). For pull-down assay, 5 mg of each purified proteins were incubated with 30 ml glutathione agarose beads in a buffer containing 25 mM Tris-HCl (pH 7.5), 100 mM NaCl and 1 mM DTT for 1 hr. The beads were washed seven times with the washing buffer containing 25 mM Tris-HCl (pH 7.5), 100 mM NaCl, 1 mM DTT and 0.1% Trition-X 100. The bound protein was eluted with elution buffer containing 25 mM Tris-HCl (pH 7.5), 100 mM NaCl, 1 mM DTT and 15 mM GSH. Immunoblot was performed with the corresponding antibodies.

### Co-immunoprecipitation assay

The Arabidopsis protoplasts prepared as reported^34^ were transformed with the indicated plasmids, and then cultured for 12 hours at 22 °C. Total protein was extracted for Co-IP assay with the extraction buffer containing 50 mM HEPES [pH 7.5], 150 mM KCl, 1 mM EDTA, 0.5% Trition-X 100, 1 mM DTT, proteinase inhibitor cocktail. For anti-FLAG IP, total protein was incubated with 50 μL agarose conjugated anti-FLAG antibody (Sigma) for 4 hr. and washed seven times with washing buffer [50 mM HEPES (pH 7.5), 150 mM KCl, 1 mM EDTA, 0.5% Trition-X 100, 1 mM DTT]. The bound protein was eluted with 60 μL 0.5 mg/mL FLAG peptides for 1 hr. The protein was separated by SDS-PAGE and subjected to immunoblot by anti-HA and anti-FLAG antibodies. For anti-HA IP, the protein was incubated with 50 mL agarose conjugated anti-HA antibody (Thermo) for 4 hr. After washing, the bound protein was eluted with 60 μL 0.5 mg/ml HA peptide for 1 hr. The protein was separated by SDS-PAGE and detected by anti-HA and anti-FLAG immunoblot. For anti-GFP IP, the protein was incubated with 50 μL agarose conjugated anti-GFP antibody (Thermo) for 4 hr. After washing, the protein was separated by SDS-PAGE and detected by anti-GFP, anti-HA and anti-FLAG immunoblot.

### Whole- Mount Clearing of Embryos

The method for phenotypic analysis of mutant embryos was described previously^35^.

### Accession numbers

Sequences from this article can be found in the GenBank/EMBL or Arabidopsis Genome Initiative database by the following accession numbers: *AGB1* (*At4G34460*), *AGG1* (*At3G63420*), *AGG2* (*At3G22942*), *GPA1* (*At2G26300*), *MPK6* (*At2G43790*), *MPK3* (*At3G45640*), *MPK4* (*At4G01370*), *MKK4* (*At1G51660*), *MKK5* (*At3G21220*), *YDA* (*At1G63700*).

## Electronic supplementary material

Supplementary Information
