# Supplementary material for: The integration of Gβ and MAPK signaling cascade in zygote development
Source: Sci Rep. 2017 Aug 18;7:8732. doi: 10.1038/s41598-017-08230-4 (PMC5562876; doi:10.1038/s41598-017-08230-4)

The integration of Gβ and MAPK signaling cascade in zygote development

Guo-Liang Yuan[1,2], Hong-Ju Li[1,*] and Wei-Cai Yang[1,*]

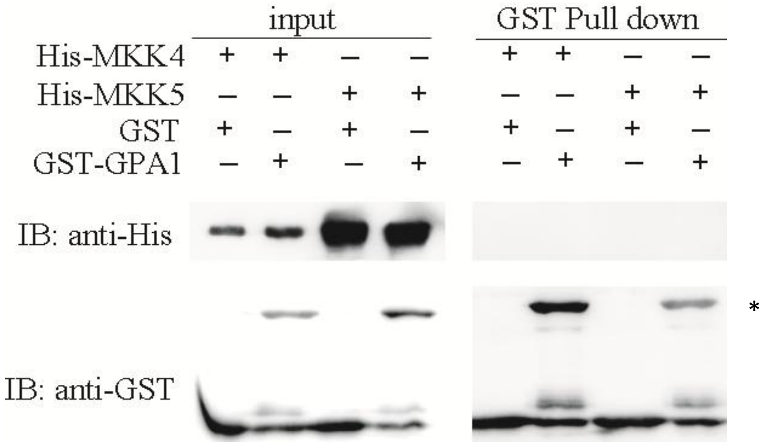

Figure S1. The interaction between MKK4/5 and GPA1. GPA1 does not interact with MKK4/5 in pull-down assay. The fusion proteins are purified from *E.Coli* and subjected to *in vitro* pull-down assay. Asterisk, target protein.

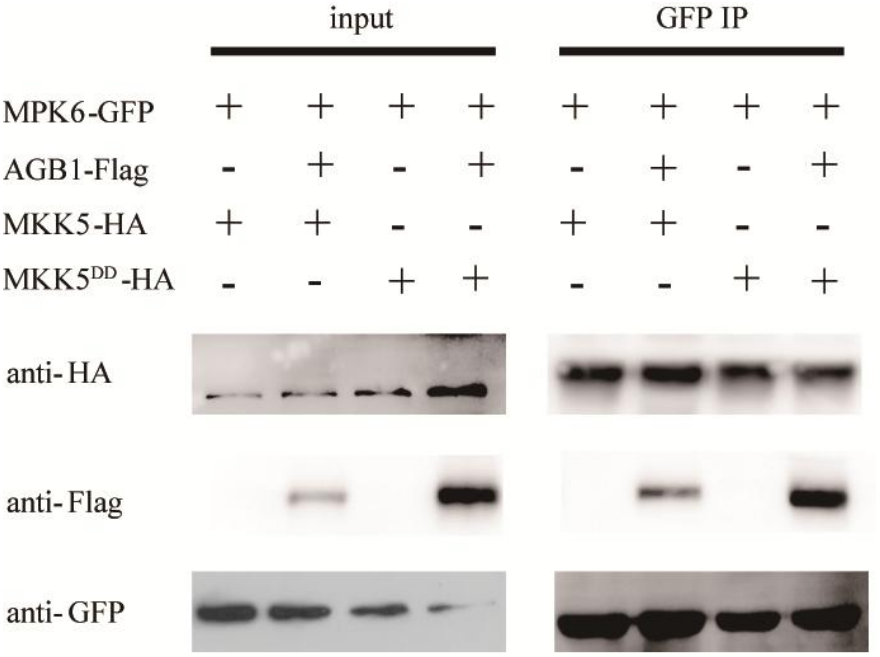

Figure S2. The interaction between MKK5/5^{DD} and MPK6. AGB1 does not promote the interaction between MKK5/5^{DD} and MPK6 when AGB1 was co-expressed with MKK5/5^{DD} and MPK6 in Arabidopsis protoplasts. MKK5^{DD}(T215D/S221D): constitutively active MKK5.

Full blot of Figure 1K

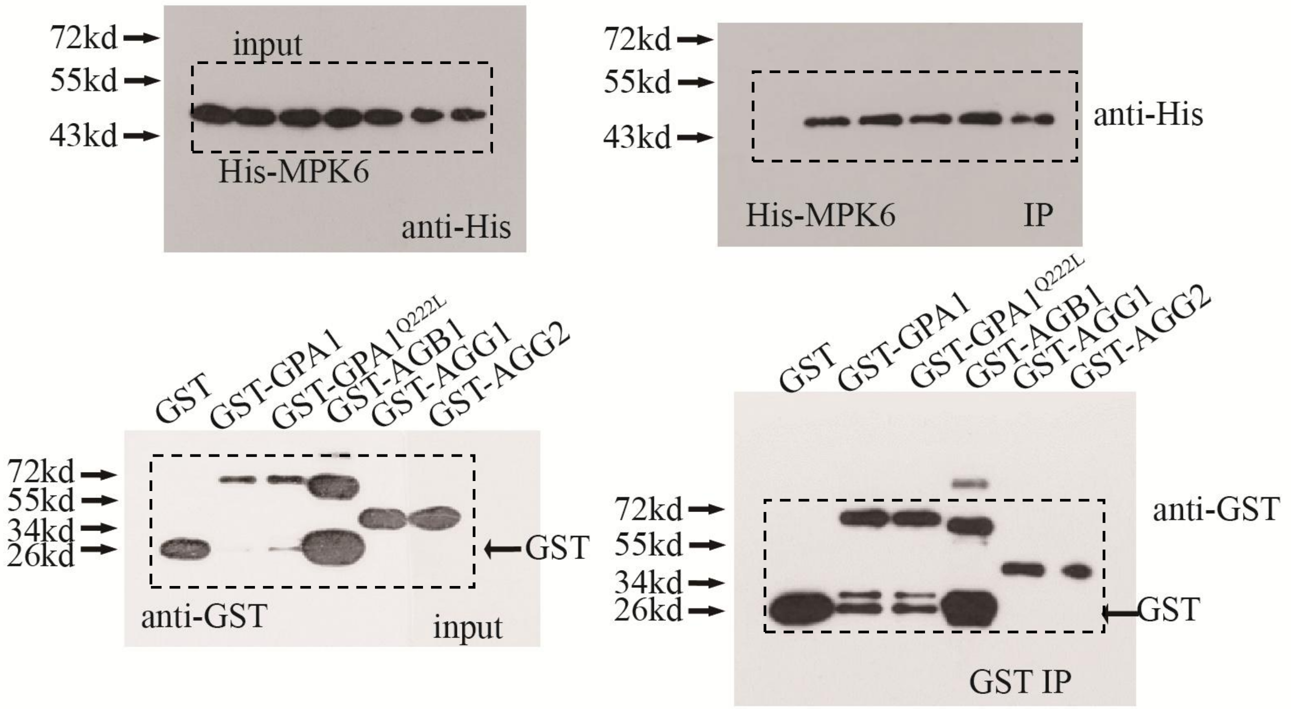

Full blot of Figure 1L

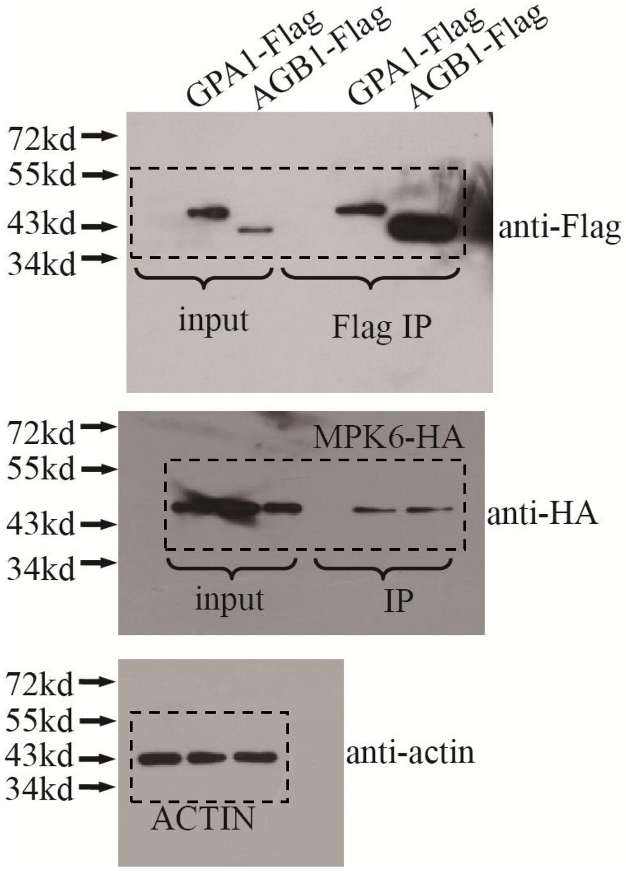

Full blot of Figure 1M

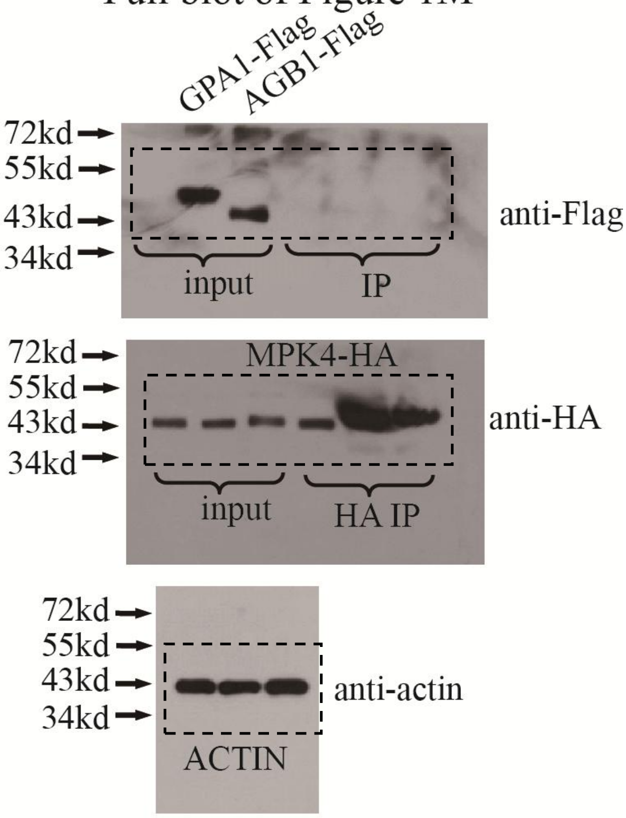

Full blot of Figure 3C

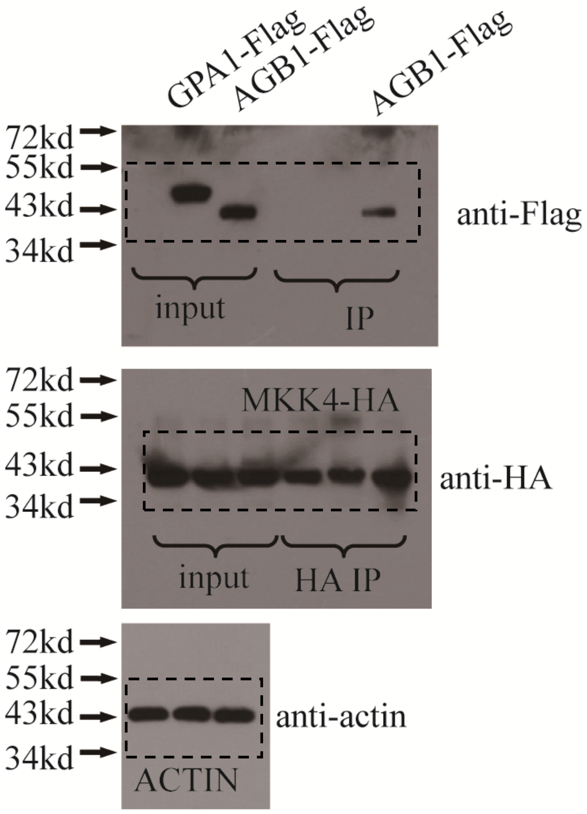

Full blot of Figure 3D

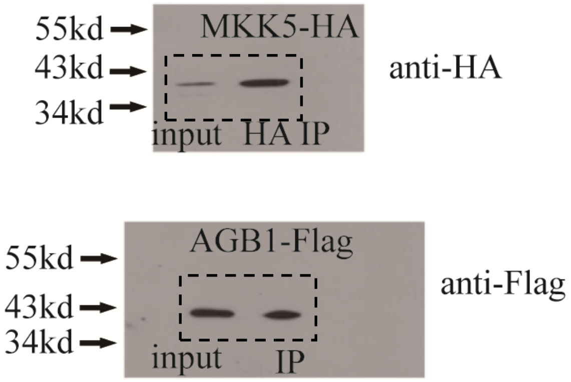

Full blot of Figure 3E

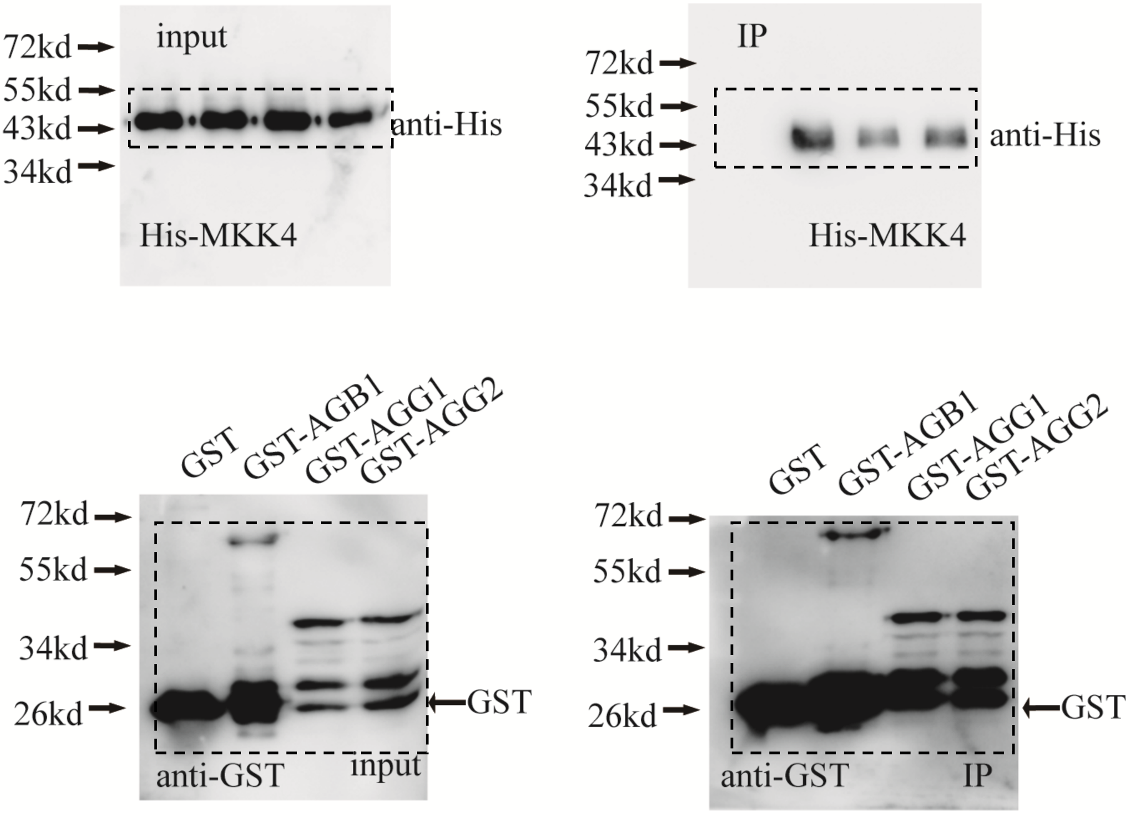

Full blot of Figure 3F

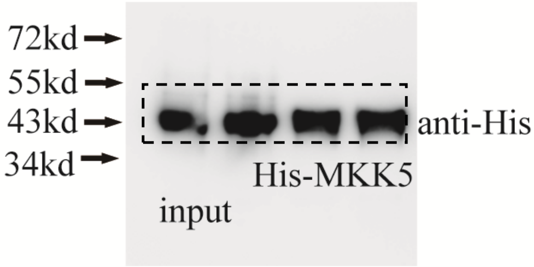

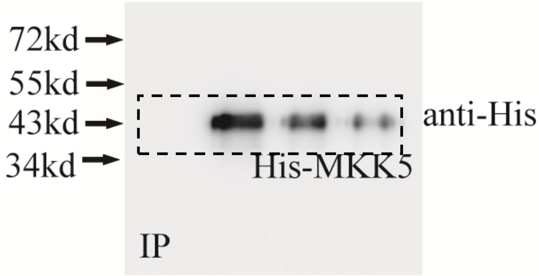

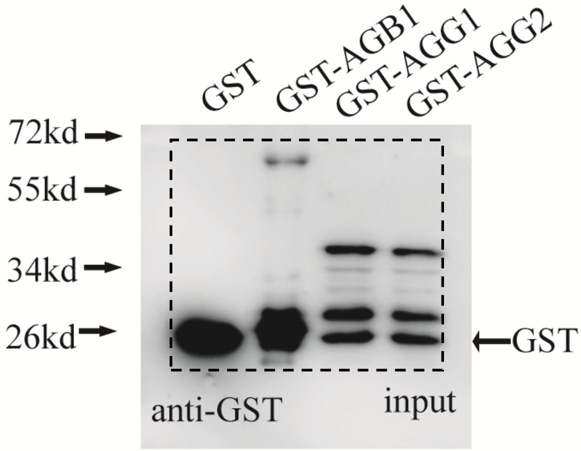

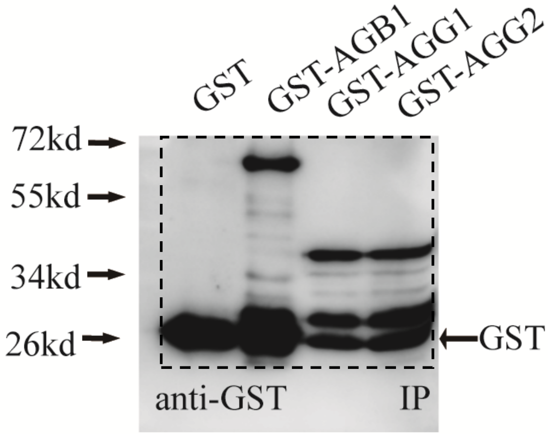

Full blot of Figure 4L

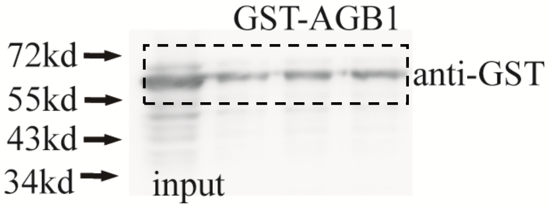

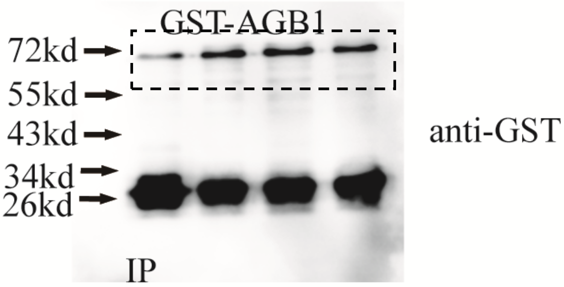

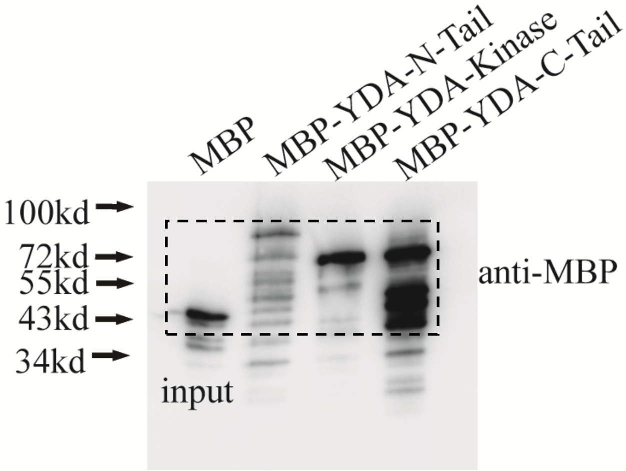

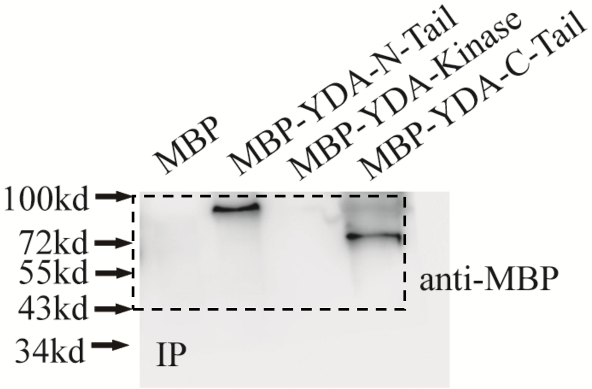

Full blot of Figure 4M

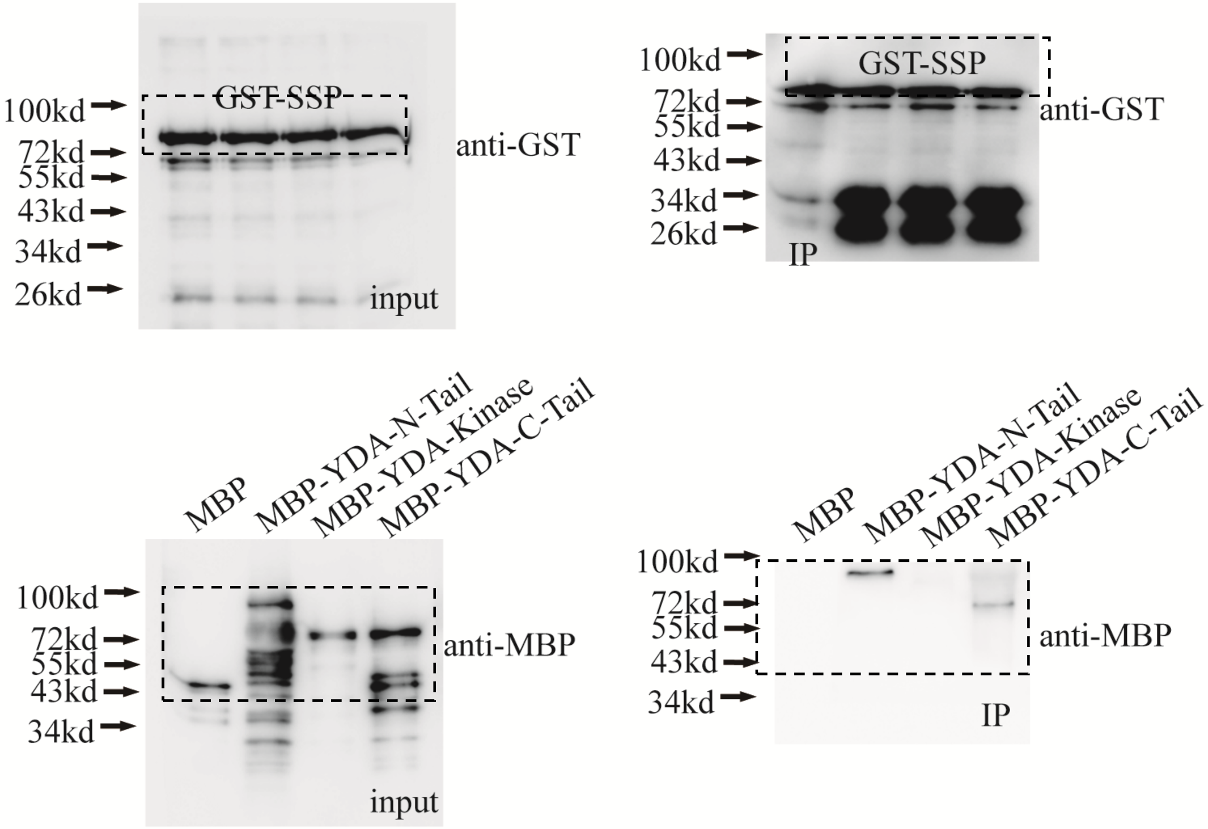

Full blot of Figure 4N

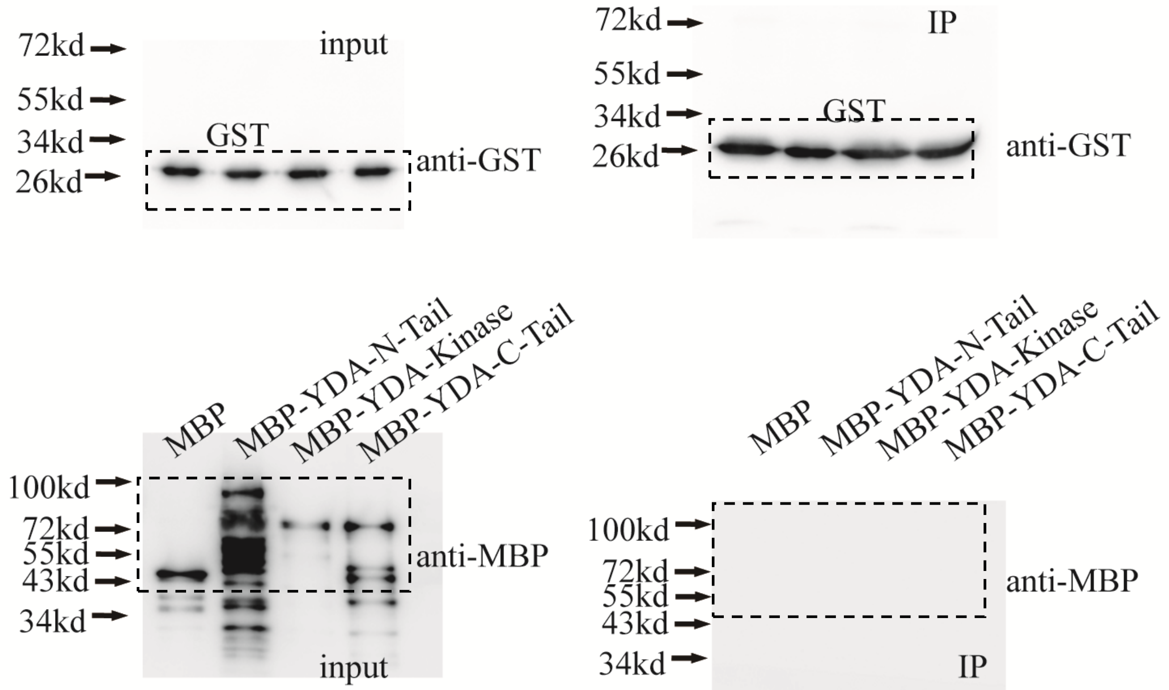

Full blot of Figure S1

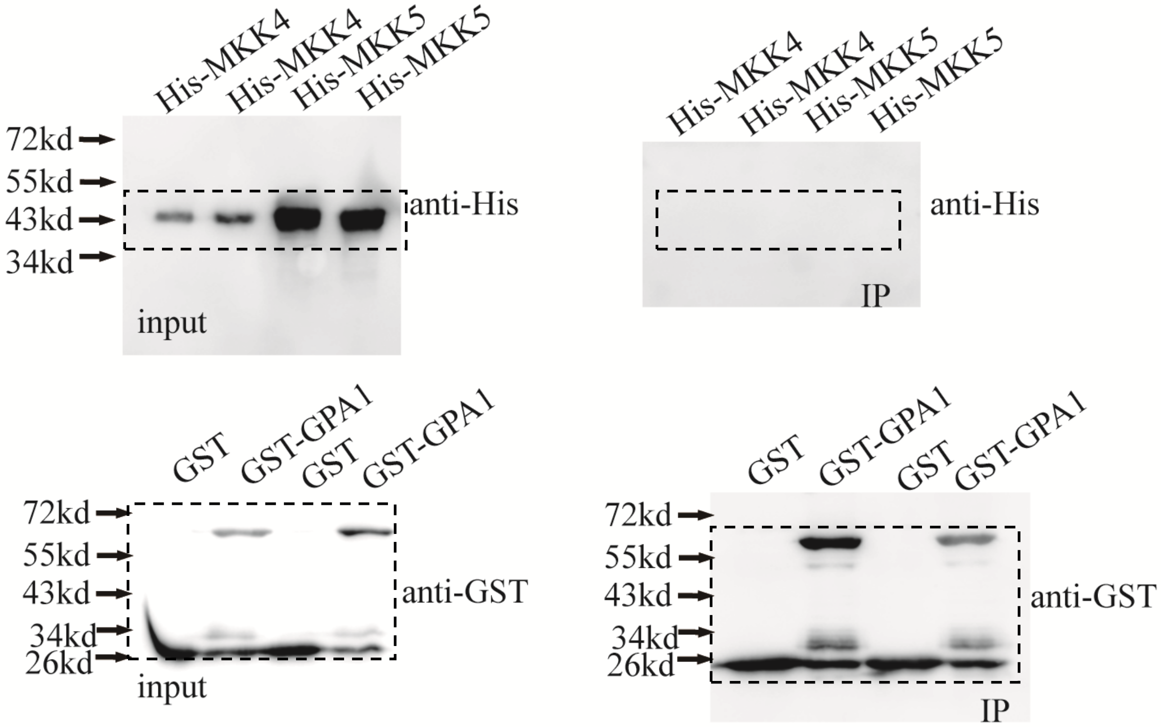

Full blot of Figure S2

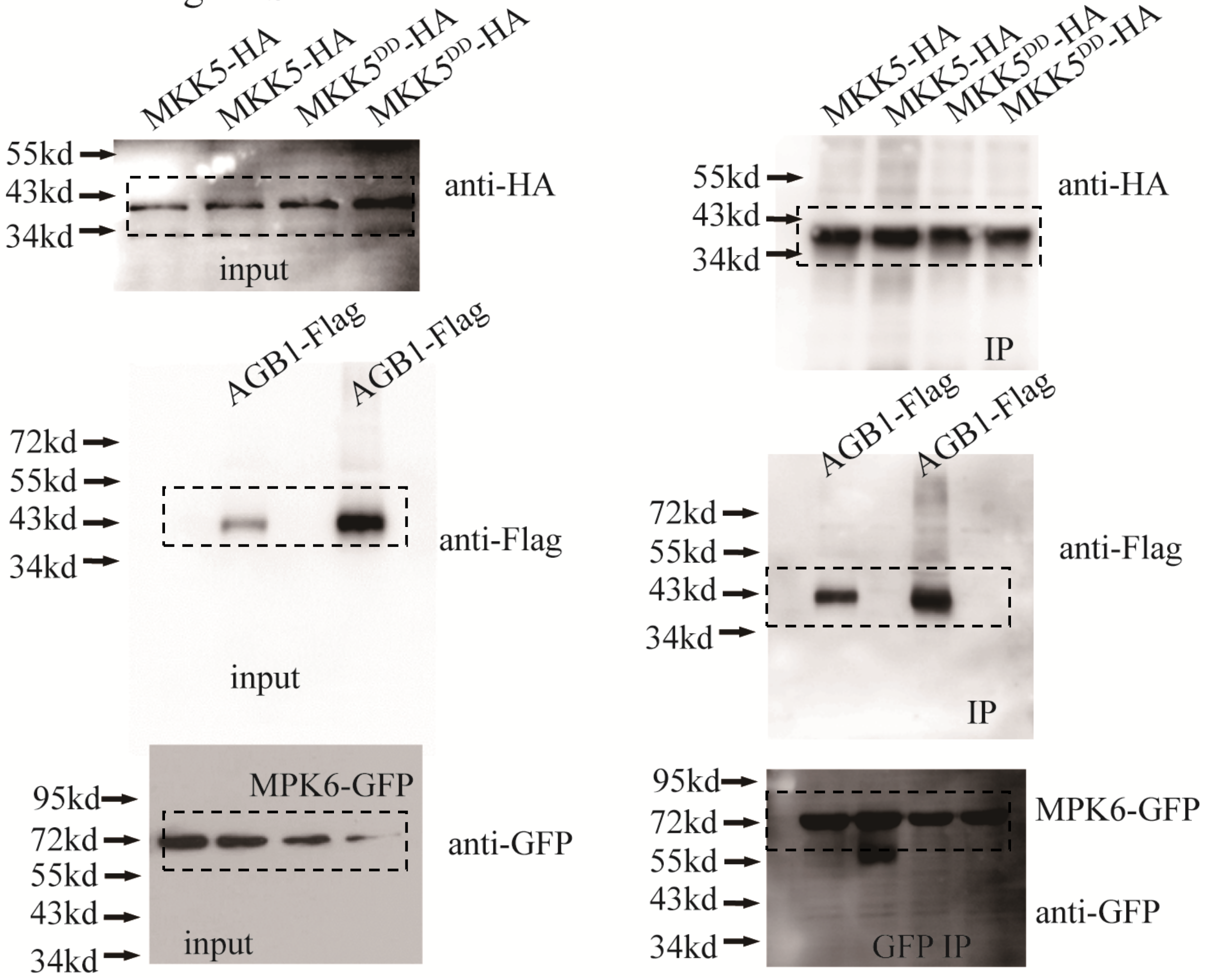

Supplement: Supplementary file 1 — Supplementary Information [file 41598_2017_8230_MOESM1_ESM.pdf]
